# The complete mitochondrial genome of epitokous *Nereis* species (Phyllodocida, Nereididae) from Dok-do, Korea

**DOI:** 10.1080/23802359.2017.1390419

**Published:** 2017-10-18

**Authors:** Hana Kim, Hyun Ki Choi, Hyung June Kim, Gi-Sik Min

**Affiliations:** a Department of Taxonomy and Systematics, National Marine Biodiversity Institute of Korea, Seocheon-gun, Chungcheongnam-do, Republic of Korea;; b Department of Biological Sciences, Inha University, Incheon, Republic of Korea

**Keywords:** Complete mitogenome; *Nereis*, nereididae, polychaete

## Abstract

The mitogenome sequence of the epitokous *Nereis* species (Phyllodocida, Nereididae) was determined first in the genus *Nereis*. The complete mitogenome is 15,667 bp in length, containing 13 protein-coding genes, two ribosomal RNAs, 22 transfer RNAs and a control region, and their gene order and structure is identical to those of other nereidid species. The mitogenome consists of 33.5% A, 20.0% C, 13.3% G, 33.1% T, showing a high content of A + T similar to the other phyllodocid polychaetes. These results will be useful for inferring the phylogenetic relationships among the members of Nereididae within the phyllodocids.

The metamorphoses of the nereid worms are known as a typical strategy for the reproduction, called the epitoky, and sexually mature nereid worms show various morphological modification (Hébert-Chatelain et al. 2008). This metamorphosis causes a taxonomic confusion due to the modification of key morphological characteristics. The molecular analysis has been widely used to solve the taxonomic problem on the morphological vagueness in as the epitokous specimens (Bucklin et al. 2011). As a result of the molecular analysis, our specimens showing epitokous form were turned out to be the species belonging to the genus *Nereis* Linnaeus 1758, but their species-level information is doubtful because of lacking of the mitochondrial sequences of *Nereis* species previously announced from GenBank. In this study, we determined the complete mitochondrial genome (mitogenome) of an epitokous *Nereis* species, and it will be useful information in the further study of the molecular taxonomy of *Nereis* species.

Specimens of the present study were collected from the subtidal zone of Dok-do Island in the East Sea (Sea of Japan) of Korea using light traps. The voucher specimens were deposited in National Marine Biodiversity Institute of Korea (MABIK NA00146040-00146044). The genomic DNA was isolated from the muscle tissue and the mitogenome sequences were analyzed by application of Illumina Hiseq2000 sequencing platform (Macrogen, Seoul, Korea). Then, we confirmed the mitogenome sequences of *Nereis* sp. by the Sanger method using each universal primer sets regarding the partial *cox1*, *cox3*, *cytb*, *nad4* and 16S ribosomal RNA (rRNA) genes. The sequences were assembled and annotated in comparison with the previously reported mitogenome sequences of nereidid species (Boore 2001; Kim et al. 2015, 2016) using Geneious 9.1.8 (Kearse et al. 2012). The phylogenetic tree was constructed using MEGA6 (Tamura et al. 2013).

The complete mitogenome of *Nereis* sp. (GenBank accession number MF960765) is 15,667 bp in length, containing 13 protein-coding genes (PCGs), two rRNAs, 22 transfer RNAs (tRNAs) and a control region (CR). All genes are encoded on the H-strand and their order and structure in the mitogenome are identical to those of other nereidid species. The overall base composition of *Nereis* sp. is 33.5% A, 20.0% C, 13.3% G, 33.1% T, showing a high A + T content (66.7%) similar to the other phyllodocid polychaetes. All PCGs use ATG as an initiation codon. Nine PCGs (*cox2, atp8, nad6, cytb, atp6, nad4l, nad5, nad3* and *nad2*) had TAA and one (*cox3*) had TAG as the stop codon, while three (*cox1*, *nad5* and *nad1*) had an incomplete stop codon, T. The lengths of tRNA genes range from 53 to 66 bp and all tRNAs have the typical clover leaf structure except tRNA^Arg^, tRNA^Ser^_UCN_ and tRNA^Ser^_AGN_. The three tRNAs have a reduced DHU arm.

To confirm the molecular phylogenetic position of *Nereis* sp., we conducted the neighbour-joining analysis with the K2P model in MEGA6 using concatenated sequences of 13 PCGs. As a result, *Nereis* sp. was grouped within Nereididae and is closely related to *Platynereis dumerilii* with high bootstrap values of more than 98% (Figure 1).

**Figure 1.**
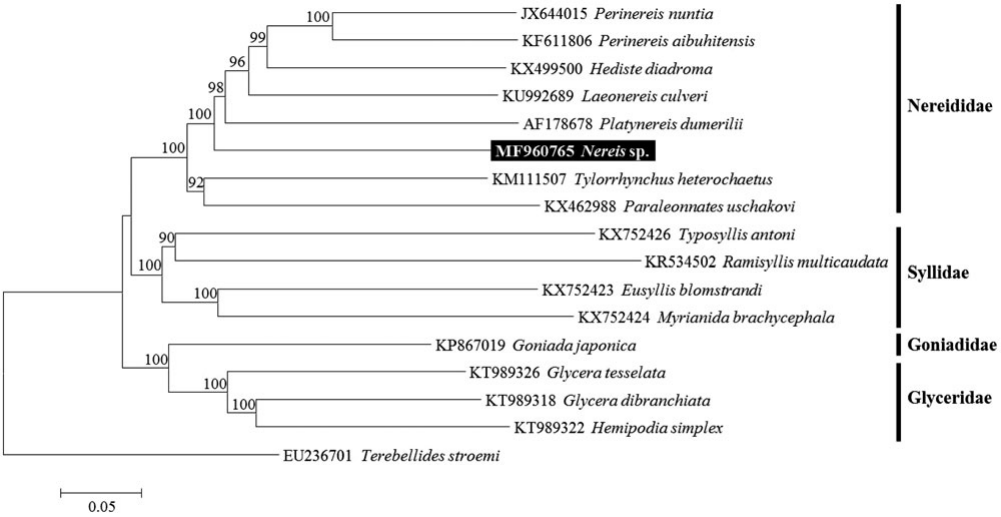
Neighbour-joining (NJ) tree based on the mitogenome sequences of eight nereidid species including *Nereis* sp. with eight other related species in Phyllodocida. *Terebellides stroemi* derived from Terebellida was used as outgroup for tree rooting. Numbers above the branches indicate NJ bootstrap values from 1000 replications.
